# The Effects of Secondary Oxides on Copper‐Based Catalysts for Green Methanol Synthesis

**DOI:** 10.1002/cctc.201601692

**Published:** 2017-04-06

**Authors:** James S. Hayward, Paul J. Smith, Simon A. Kondrat, Michael Bowker, Graham J. Hutchings

**Affiliations:** ^1^ Cardiff Catalysis InstituteMain Building, Cardiff UniversityPark PlaceCardiffCF10 3ATUK; ^2^ Research Complex at Harwell, Rutherford Appleton LabHarwell OxfordOxfordOX11 0FAUK

**Keywords:** CO_2_, copper, methanol synthesis, oxides, supercritical liquids

## Abstract

Catalysts for methanol synthesis from CO_2_ and H_2_ have been produced by two main methods: co‐precipitation and supercritical anti‐solvent (SAS) precipitation. These two methods are compared, along with the behaviour of copper supported on Zn, Mg, Mn, and Ce oxides. Although the SAS method produces initially active material with high Cu specific surface area, they appear to be unstable during reaction losing significant amounts of surface area and hence activity. The CuZn catalysts prepared by co‐precipitation, however, showed much greater thermal and reactive stability than the other materials. There appeared to be the usual near‐linear dependence of activity upon Cu specific area, though the initial performance relationship was different from that post‐reaction, after some loss of surface area. The formation of the malachite precursor, as reported before, is important for good activity and stability, whereas if copper oxides are formed during the synthesis and ageing process, then a detrimental effect on these properties is seen.

## Introduction

In recent years the continuing rise in atmospheric anthropogenic carbon and the ensuing effects that this engenders on the climate have led to increasing efforts towards capture, sequestration, and utilisation of carbon dioxide. Given a general societal trend towards greener energy and fuel sources, the utilisation of CO_2_ as an abundant carbon source has become more and more relevant. The hydrogenation of CO_2_ to produce methanol for use as both a fuel and a chemical precursor is one possible route to this goal. The concept of a cycle using CO_2_ and methanol in such a manner is generally attributed to Olah et al.,^1^ and is referred to as the anthropogenic carbon cycle. One of the attractive facets of this method is the possibility of a truly green fuel; CO_2_ can be captured from sources such as power stations and combined with hydrogen generated from a renewable source, such as electrolysis of water where the electricity is supplied from solar power. The synthesis of green methanol in this way can be regarded as a way of storing H_2_, effectively for storing renewable energy chemically, in addition to the production of a fuel.

Global methanol production is in the region of 80 Mt per annum.^2^ Industrially, methanol is produced from a mixture of carbon monoxide, carbon dioxide, and hydrogen (syngas) at elevated pressures and moderate temperatures. It has been shown that carbon dioxide is the carbon source at the molecular scale, producing methanol and water. Carbon monoxide is present to convert the water produced into CO_2_ and H_2_ via the water‐gas shift reaction. These reactions are represented by Equations (1), (2):

(1) $$\mathrm{CO}{}_{2}+3H{}_{2}\to \mathrm{CH}{}_{3}\mathrm{OH}+H{}_{2}O\;\;\left(\mathrm{methanol}\;\mathrm{synthesis}\right)$$

(2) $$\mathrm{CO}{}_{2}+H{}_{2}\to H{}_{2}O+\mathrm{CO}\;\;\;\left(\mathrm{reverse}\;\mathrm{water}\text{-}\mathrm{gas}\;\mathrm{shift}\right)$$

The current industrial synthesis is from a syngas, deriving from fossil fuels, which has a mix of CO and CO_2,_ but the main synthesis route is as in Equation (3), with little water production:

(3) $$\mathrm{CO}+2H{}_{2}\to \mathrm{CH}{}_{3}\mathrm{OH}$$

However, the reaction studied herein differs from this system in that there is no CO present, since we are testing the possibility of using recaptured CO_2_ and renewable hydrogen, as opposed to fossil fuel generated syngas. This means that without CO being present to enable overall water‐gas shift, our system will contain a significantly higher proportion of water vapour than a system running syngas.

The catalysts used for this reaction are composed of copper, zinc, and alumina, and are based on the catalysts originally designed by ICI during the 1960s.^3^, ^4^ The optimisation of these catalysts was performed long before modern techniques made it possible to understand the fundamentals of the active sites and reaction mechanism. In recent years there have been increasing numbers of studies into these fundamental aspects of this catalysis,^5^, ^6^ with the focus generally tending towards the simpler binary system of Cu/ZnO. From this has risen the consensus that the methanol productivity is strongly correlated with the specific copper surface area of a catalyst,^7^, ^8^ and that other factors such as the oxidation state of the copper,^9^ and the copper–zinc interaction,^10^, ^11^ also have an effect.

The optimised industrial catalyst is granted excellent copper surface area by way of the structure of the precursor material from which it is derived. Such catalysts are synthesised by co‐precipitation of metal nitrate salts with sodium carbonate to produce a hydroxycarbonate precursor phase, which is then calcined to form CuO and ZnO. It is known that the most active catalysts are formed from a precursor consisting predominantly of zinc‐substituted malachite phases. When properly prepared, the specific structure of the final catalyst is defined by this precursor phase, leading to a material with the desired high copper surface area and good copper–zinc interaction.^12^

Whilst copper comprises the active metal in the catalysts, the role of the zinc is less clear,^13^ and as such there have been many investigations into copper‐based catalysts with alternative secondary metal oxides such as magnesia,^14^ ceria,^15^, ^16^ and zirconia.^17^, ^18^ Of particular note are studies that show Cu/MgO catalysts as having higher copper surface areas, and yet having lower methanol activity,^14^ which runs counter to the accepted stance of copper surface area being directly linked to such activity. The issue seems to stem from the nature of the standard catalyst, the synthesis of which has been highly optimised in terms of pH, temperature and ageing times. Such catalysts are generally precipitated in the range of pH 6–7, and have been shown to lose activity if precipitated at higher pH ranges.^12^ However, the precipitation of magnesium nitrate requires a pH in the region of 9. Thus it is difficult to deconvolute whether the negative effect on activity is an artefact of the substituted oxide or the pH of synthesis.

Supercritical anti‐solvent (SAS) precipitation presents an interesting way to approach this problem, as the procedure rapidly precipitates material without the need for a base. It has been shown that this method can produce copper–zinc catalysts with high copper surface area, and that these catalysts are active for methanol synthesis and water‐gas shift reactions.^19^ A wide range of materials can be precipitated in this manner, in all cases without the requirement of a specific precipitating agent. This allows us to sidestep the need for specific pH ranges found in co‐precipitation, allowing us to remove it as a factor.

In this study we report the changes in methanol synthesis activity for copper catalysts synthesised with various secondary oxides using both co‐precipitation and SAS techniques. The changes were monitored through reactivity measurements, and through assessment of the copper surface area and particle size both before and after exposure to reaction conditions. Through this we hope to discover what factors result in activity loss in the co‐precipitated catalysts, and to investigate which metal oxides are capable of producing active, stable catalysts when the negative effects of high‐pH co‐precipitation are removed.

## Results and Discussion

This study was conducted using Cu/M_*x*_O_*y*_ catalysts, where M=Mg, Zn, Mn, or Ce. Based on the results of previous studies a molar ratio of 70:30 between copper and the secondary oxide was chosen. This amount has been shown to be close to the limit of incorporation of zinc into a malachite structure,^20^ and as such was used as a standard to which the other catalysts were held.

### Co‐precipitated catalysts

The catalysts were prepared as described in the Experimental Section. The surface areas of the materials produced are shown in Table 1.

**Table 1:** Co‐precipitated catalyst details.

			Surface areas [m^2^ g^−1^]	
Catalyst	Synth*e*sis pH	Precursor colour	Precursor	Calcined
CuZn‐CP‐1	6.5	blue–green	121	119
CuZn‐CP‐2	9	blue–green	120	117
CuZn‐CP‐3	10	blue–green	105	102
CuMn‐CP	6.5	green–brown	116	109
CuMg‐CP	9	dark blue	128	125
CuCe‐CP	10	dark brown	85	77

**Figure 1:**
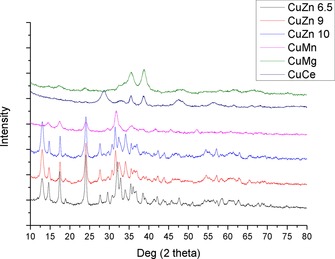
XRD patterns of co‐precipitated catalyst precursors.

X‐ray diffraction (XRD) of the precursor phases revealed patterns consistent with those of malachite^21^ in the cases of all CuZn‐CP catalysts, as shown in Figure 1. The other precursors showed broader diffraction peaks. CuMg‐CP and CuCe‐CP show two major diffraction peaks consistent with CuO, with CuMg‐CP having low, broad peaks consistent with malachite. The small peak at 33° in CuCe‐CP may be residual malachite, and this material also shows broad peaks at angles consistent with CeO_2_. Based on these observations it would seem that the hydroxycarbonate phase is formed initially in all cases, as evidenced by the blue coloured material often reported in such cases. The colour change towards green in the CuZn and CuMn catalysts can be attributed to the formation of the malachite phase, whereas the darkening in colour of the CuMg and CuCe catalysts can be attributed to the formation of copper oxide phases. This cannot solely be attributed to the effects of the pH, as the CuZn catalysts prepared at higher pH do not show these phases. Therefore, this would seem to be an effect of the oxide, although possibly this is in combination with the elevated pH.

The work of Fujita et al.^22^, ^23^ showed that a calcination temperature of 330 to 350 °C is sufficient to form the final catalysts. Based on this, all catalysts were calcined at 330 °C for 3 h in flowing air, with a thermal ramp rate of 5 °C min^−1^. The catalysts were uniformly brown after this calcination step, with the exception of CuCe‐CP, which presented a slightly grey hue.

**Figure 2:**
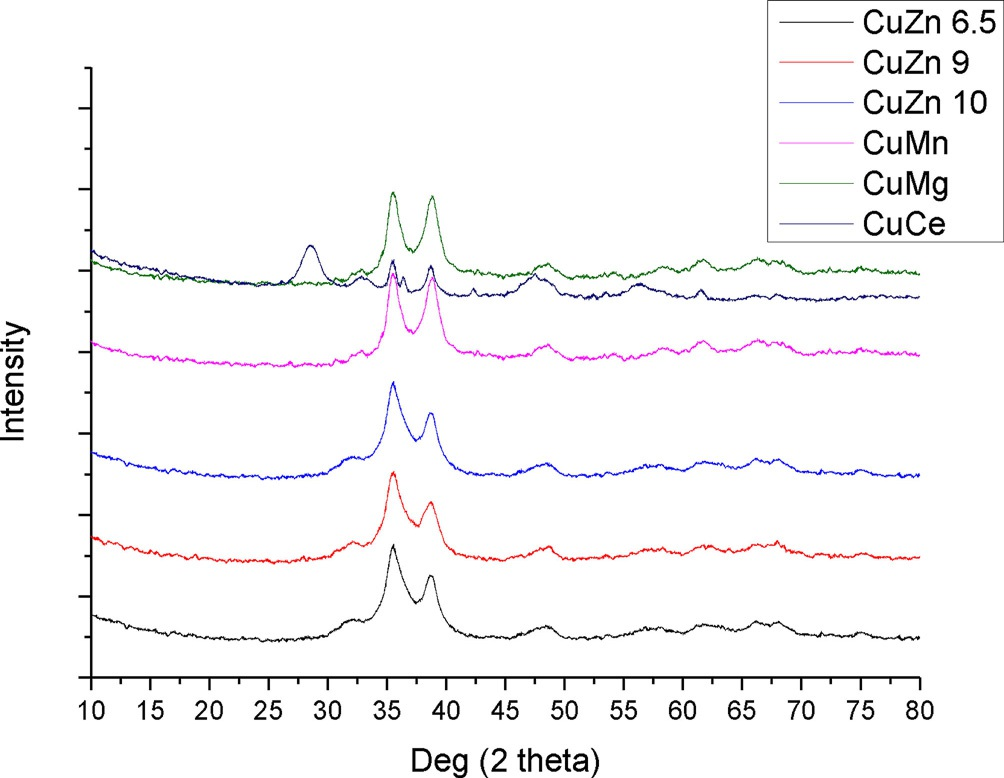
XRD patterns of co‐precipitated catalysts after calcination.

XRD of the calcined catalysts gave similar patterns for all the materials except for CuCe‐CP (Figure 2). All had peaks consistent with copper oxide, but with the CuCe‐CP material having additional peaks consistent with CeO_2_. With the exception of the appearance of a small, sharp peak at 36°, the precursor and calcined versions of CuCe‐CP are very similar. The precursor and calcined versions of CuMg‐CP are also highly similar, with the calcined version losing the small, broad peaks associated with malachite. This is consistent with copper oxide already being formed during drying in these materials. The surface areas of the materials after calcination decreased by less than 10 % from the values found in the precursors.

The catalyst samples were tested for methanol synthesis as described in the Experimental Section, and the only significant products seen were CO and methanol. The CuZn catalysts generally appeared to undergo a strong initial deactivation, but were stable after 3 h. The other secondary oxides displayed varying behaviour, which will be discussed below. Of the co‐precipitated catalysts, copper–zinc showed the highest activity towards methanol production, and also showed the lowest amount of deactivation (Table 2). A trend amongst the copper–zinc catalyst was also evident; increasing pH lowered CO_2_ conversion and methanol selectivity, while those at lower pH preparation tended to show a lower degree of deactivation. CuZn 6.5‐CP lost only 5 % CO_2_ conversion from 1–8 h, compared with 7 % for CuZn 9‐CP and 10 % for CuZn 10‐CP. CuMn‐CP and CuMg‐CP had similar, if not higher, copper surface areas than the CuZn‐CP catalysts before reaction, but were not as active. CuMg‐CP gave good CO_2_ conversion and excellent methanol selectivity, but continued to deactivate after 3 h, stabilising after 6 h. CuMn‐CP appears to have gained activity over time, but a full time on‐line reaction study showed a slightly more complicated effect. CuMn‐CP started with the low activity shown above, and appeared to immediately start deactivating. However, after about 2 h, it began to show a marked increase in activity over the next hour, with both CO_2_ conversion and methanol selectivity rising rapidly to approximately 6.3 % conversion and 63 % selectivity. It maintained this activity for about 2 h before undergoing a rapid deactivation. Of particular interest is that the overall CO production rate changed only slightly during this time, implying that the increased CO_2_ conversion was primarily driven by a large increase in methanol selectivity, and that the deactivation occurred in the reverse manner. This would seem to indicate that species or active sites are briefly formed on CuMn catalysts that are highly active, but highly unstable. A repeat of this test over a longer time period (16 h) showed that the deactivation continued beyond 8 h, with the material having apparently stabilised after about 11 h, at which time it displayed CO_2_ conversion of <1 %. CuCe‐CP deactivated steadily, stabilising only in the final hour of testing. Whilst it was the only catalyst to increase methanol selectivity steadily, it does not seem to be a viable catalyst due to high deactivation and low activity.

**Table 2:** Catalytic activity and copper surface areas of co‐precipitated catalysts.

	CO_2_ conversion		Selectivity				Cu SSA	
	[%]		MeOH [%]		CO [%]		[m^2^ g^−1^]	
Catalyst	1 h	8 h	1 h	8 h	1 h	8 h	Pre	Post
CuZn‐CP‐1	4.5	4.3	55	53	45	47	21	15
CuZn‐CP‐2	4.1	3.7	53	51	47	49	20	12
CuZn‐CP‐3	3.5	3.2	32	30	68	70	18	10
CuMn‐CP	2.8	3.5	38	54	62	46	18	5
CuMg‐CP	3.9	1.1	69	40	31	6	24	7
CuCe‐CP	2.3	0.8	38	61	62	39	15	3

**Figure 3:**
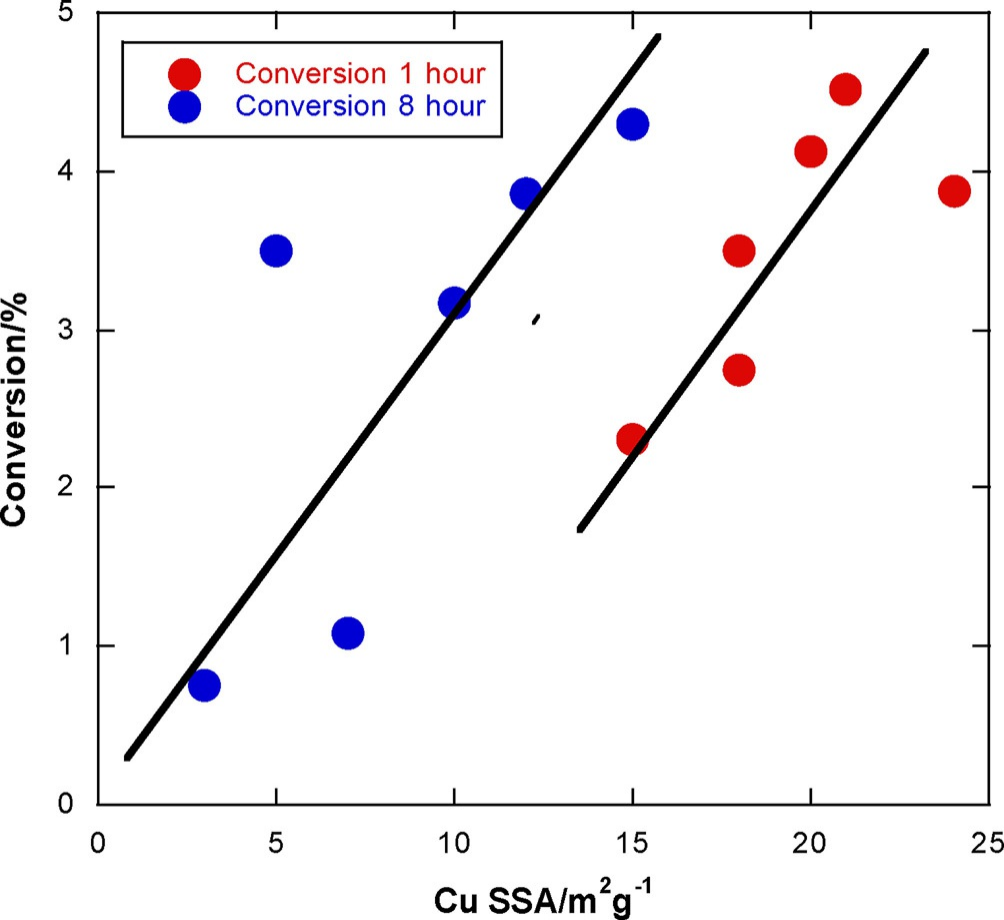
The dependence of catalyst activity on the Cu metal surface area for co‐precipitated catalysts. The lines are a guide for the eye. The red data points are for initial conversion and pre‐reaction metal area, whereas the blue data points relate the final conversion and metal surface area measured post‐reaction.

The relationship of the copper surface areas to activity is interesting (Figure 3 and Table 2). Initial copper surface areas appear to match trends in the activity quite well, with the notable exception of CuMg‐CP. This catalyst possesses higher copper surface area than any of the others, and yet has lower activity. However, if one considers the post‐reaction copper surface areas, there is a more evident trend. Here, the higher surface areas correspond to higher activities with the exception of the CuMn‐CP sample. However, the CuMn‐CP catalyst was observed to be deactivating rapidly at the termination of the reaction, and the depressurising and cooling steps before recovery of the catalyst take approximately an hour. It may be that the CuMn‐CP catalyst continued to deactivate through this time. It appears that, although conversion has generally diminished, the intrinsic per site activity has increased after 8 h running, evident in the data of Figure 3. It is likely that this is due to a morphology change of the Cu particles, perhaps such that the Cu−ZnO interaction is not lost as much as the Cu surface area.

Thus, the value of the copper–zinc catalysts would appear to be a combination of high initial copper surface area and their ability to better retain this surface area during reaction conditions.

### Supercritical anti‐solvent (SAS) precipitation catalysts

Supercritical anti‐solvent precipitations were carried out as described below and produced very fine powder, which was either blue or green depending on the secondary oxide (Table 3).

**Table 3:** Supercritical anti‐solvent precipitation catalyst details.

		Surface areas [m^2^ g^−1^]	
Catalyst^[a]^	Precursor colour	Precursor	Calcined
CuZn‐SAS	light blue	157	110
CuMn‐SAS	light green	100	72
CuMg‐SAS	light blue	150	144
CuCe‐SAS	light green	112	69

**Figure 4:**
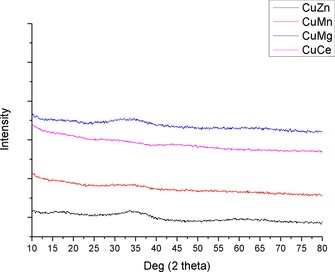
XRD patterns for SAS catalyst precursors.

XRD analysis on the precursor phases showed them to be highly amorphous/nanoparticulate (Figure 4). It is difficult to thus draw any conclusions about the materials formed, but these observations are in line with those reported for the synthesis of supercritically prepared georgeite^19^, ^24^ It is possible that the other oxides form similar amorphous materials as an effect of the extremely rapid precipitation step found in SAS precipitations.

**Figure 5:**
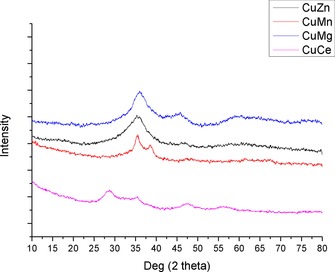
XRD patterns of SAS catalysts after calcination.

XRD analysis on the calcined SAS materials (Figure 5) showed a number of similarities to the CP materials. CuCe shows a small diffraction peak in the region of copper(II) oxide, and shows broad reflections consistent with the presence of CeO_2_. The other materials all appear to be copper oxide, as was observed in the CP materials. However, whereas the CP materials all displayed a distinctive double peak, the CuZn‐ and CuMg‐SAS show a single, broader reflection. This is indicative of smaller crystal domains in the material, which is likely to be an effect of the highly amorphous precursor being unable to generate long‐range order upon calcination. Unlike the co‐precipitated catalysts, the SAS catalysts displayed a far more significant loss of surface area upon calcination, with all losses being in the region of 30 %. A notable exception to this is CuMg‐SAS, which lost less than 5 %.

Catalytic testing and copper surface area measurements were carried out in an identical manner to those described for the co‐precipitated materials.

**Figure 6:**
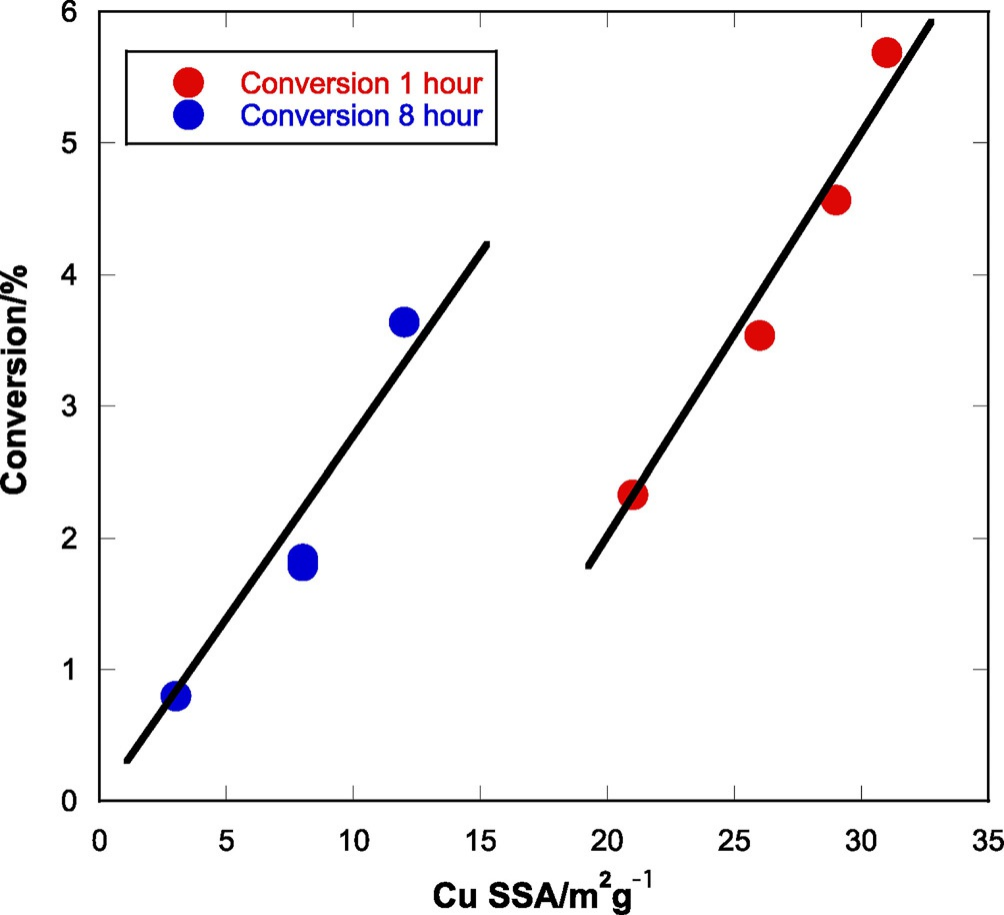
The dependence of catalyst activity on the Cu metal surface area. The lines are a guide for the eye, but indicate linear behaviour, though here the number of data points is limited. The red data points are for initial conversion and pre‐reaction metal area, whereas the blue data points relate the final conversion and metal surface area measured post‐reaction.

The reactivity behaviour of the SAS catalysts can be seen to be significantly different from that of the co‐precipitated materials, with the possible exception of the CuCe‐SAS material, which in both cases shows decreasing activity but increasing methanol selectivity (Table 4). These catalysts all displayed stronger initial deactivation than their co‐precipitated counterparts, but were all stable after 5 h. The CuZn‐SAS material is of particular interest. In keeping with evidence that increased copper surface area is directly linked to increased CO_2_ conversion (Figure 6), it is the most active catalyst when based on the results taken after 1 h. It remains active for approximately 4 h, but loses methanol selectivity as it does so. After this point, it begins to rapidly lose activity and continues to lose selectivity, stabilising after 5 h. Cu SSA measurements show a significant drop as a result of the reaction.

**Table 4:** Catalytic activity and copper surface areas of SAS catalysts.

	CO_2_ conversion		Selectivity				Cu SSA	
	[%]		MeOH [%]		CO [%]		[m^2^ g^−1^]	
Catalyst	1 h	8 h	1 h	8 h	1 h	8 h	Pre	Post
CuZn‐SAS	5.7	1.8	71	46	29	54	31	8
CuMn‐SAS	2.3	0.8	77	18	23	82	21	3
CuMg‐SAS	4.6	3.6	66	64	34	36	29	12
CuCe‐SAS	3.5	1.8	42	56	58	44	26	8

The CuMn‐SAS sample once again displayed a more complex behaviour than is suggested. Initial results shown here are at *t*=1 h, but data recorded before this point show CuMn‐SAS to be highly active, more so than CuZn‐SAS. However, it immediately deactivates, losing over 60 % of its activity in the initial hour. After 4 h it has almost completely deactivated and is predominantly selective towards the production of CO. The copper surface area measurements show a significant loss of surface area throughout the reaction, which is likely to be the cause of this deactivation. It is of note, though, that the initial activity is significantly higher than the pre‐reaction Cu SSA of the CuMn‐SAS sample would suggest. It is possible that this is a similar effect to that which was seen for CuMn‐CP, but without the induction period. The highly mixed and amorphous structure of the CuMn‐SAS could be very active initially, but then undergoes severe deactivation for the same reasons as before. This would seem to be borne out by the dramatic drop in copper surface area.

CuMg‐SAS displayed behaviour entirely contrary to the CP equivalent, proving to be the most stable of the SAS catalysts in terms of both CO_2_ conversion and selectivity. It does not appear to display such rapid deactivation, nor the switching to CO production of its CP counterpart, and after 8 h is comparable in activity to CuZn 6.5‐CP, which is due to its higher selectivity. Whilst it is shown to lose a significant amount of copper surface area, the loss is nowhere near as severe as in the cases of the other SAS materials.

## Conclusions

A number of conclusions can be drawn from the results herein. One of the initial questions was to what extent, in co‐precipitated catalysts, is he activity of copper‐based catalysts determined by the pH of precipitation and to what extent is it affected by the secondary oxide. Based on the results shown, we can say that both play a role in the activity of the catalyst. CuZn‐CP catalysts were more active than all of the other secondary metals at the equivalent pH. From the results of the CuZn materials we see that increased pH leads to decreased activity. A difference in precipitation behaviour was seen as well; whereas the CuZn‐CP catalysts form zincian malachite at all three pH values, when the zinc is replaced with magnesium or ceria at elevated pH it leads to the direct formation of copper oxides during the ageing step of the synthesis, as confirmed by XRD. The better performance of the Zn materials is probably due to the formation of this phase.

CuMn‐CP showed interesting behaviour, in that there appeared to be an induction period where the activity increased, reaching a plateau for a time before rapidly deactivating. In many ways this mirrors the findings of Helveg et al.,^25^ who showed that copper–zinc catalysts will display similar tendencies depending on the oxidising or reducing nature of the atmosphere. Although the gas mixture is highly reducing due to 60 % H_2_, the oxidising nature of the atmosphere increases with increasing steam content.^26^ As H_2_O is a by‐product of both the methanol and reverse water‐gas shift reactions which occur, it would seem that the catalyst generates an active phase which is then adversely affected by the increased water content that this improved activity engenders. This then leads to the severe deactivation seen.

Once the results of the SAS catalysts are factored in, more conclusions can be drawn. Whilst for the CP materials increased pH leads to lower activity, the SAS results show that this is not the sole determinant. The activities still do not correlate exactly with the Cu SSA, as the CuMg has a higher area than CuZn. This shows that there is indeed an additional effect from the secondary oxide beyond the simple improvement of the active metal surface area, and that the relatively lower activity of the CuMg catalysts is not only a result of the higher precipitation pH required in CP.

Further investigation of these effects are required to ascertain which properties of the secondary oxides are affecting the Cu. H_2_‐TPR could be useful to investigate the reducibility of the catalysts, and CO_2_‐TPD can be used to assess changes in the basicity of the catalysts.

The CuZn‐SAS catalyst shows a similar deactivation to that reported before, although it does not show the initial induction period. Interestingly, the CuMn catalyst appears to have very similar behaviour, although it deactivates even more swiftly. Both CuZn‐SAS and CuMn‐SAS suffer a particularly pronounced loss of Cu surface area during the reaction, with CuMn‐SAS falling to the lowest value of any tested catalyst. Based on these overall results, it would seem that Mn and Zn behave in a broadly similar manner when paired with Cu, but that Zn is the better choice due to increased stability of the supported Cu metal.

CuMg catalysts proved interesting, as they were the only instance in which the SAS material was more stable than the CP material. This is seen in both the activity data and the copper surface area data, and could be down to a number of factors. The CuMg‐CP material showed evidence of CuO formation during the initial precipitation, and whilst this material had a high copper surface area it swiftly deactivated under reaction conditions. This behaviour was not observed in the SAS material, implying that the formation of the CuO phase was not conducive to retention of the high copper surface area even though it generated a high initial value. The amorphous SAS precursor, however, led to a material more stable than its CP counterpart or any other SAS prepared material. This may be due to the properties of MgO itself, which is not reported to form strong interactions with Cu (unlike zinc) and does not have a variety of possible oxidation states (unlike manganese and ceria).

When taken as a whole, the results strongly imply that whilst the initial copper surface area is important, the ability to retain this surface area whilst under reaction conditions would appear to be key. Further, the idea that copper surface area is directly correlated to methanol activity may not be easily applicable to materials using different secondary oxides. An excellent example of this lies in the CuMg catalysts. CuMg‐CP has a higher initial copper surface area than its CuZn‐CP equivalent, but its rapid deactivation means that the post‐reaction area value shows a truer measure of its activity. The same is true of CuMg‐SAS and CuZn‐SAS. In this instance the CuMg‐SAS has the lower initial surface area, but proves to be the more active catalyst in the long run due to its stability. This focus on stability appears to be a strength of the co‐precipitated CuZn materials, which displayed the lowest amount of deactivation.

Thus, the stability of the materials, and their effectiveness as catalysts, can be attributed to a number of factors beyond initial copper surface area. The formation of the malachite phase seems to be especially important in coprecipitation; CuZn and CuMn‐CP catalysts form this phase, and were significantly more active than their amorphous SAS counterparts. This phase appears to grant a greater degree of stability to the resulting catalysts. Where materials did not form this phase, they were all found to be less stable. This effect cannot be attributed to the presence of zinc as the secondary oxide, as the CuZn‐SAS catalyst was highly unstable. By contrast, the formation of the CuO phase during precipitation was indicative of a poor catalyst.

The results obtained using the SAS‐prepared catalysts help to back up the benefits of the malachite phase, but also show that for some materials the pH is a significant factor. CuMg is a good example of this; neither the CP nor the SAS catalyst form the malachite phase, but the elevated pH led to the formation of the undesirable CuO phase during co‐precipitation. Where this phase was not observed, in the SAS material, the catalyst was far more effective. This was not the case for the CuCe materials, which were less effective regardless of preparation method. This indicates that the choice of oxide is highly relevant.

Overall, the results seem to show that when considering co‐precipitation, CuZn catalysts appear to be significantly better due to a number of benefits granted by the precursor phase. CuMn catalysts behave in a similar manner, but deactivate more rapidly. When the materials were prepared by a method which leads to a highly amorphous precursor, other oxides become viable. CuMg seems in particular to be hampered by the high pH needed for precipitation. Once this limitation was removed, it proved to be an effective catalyst. This could potentially be of use as other precipitation methods are investigated.

Another important conclusion is the apparent confirmation of the work of Hadden et al.,^27^ who suggested that the correlation between copper surface area and activity was only valid between families of catalysts prepared with similar method. This is borne out in our results, as the higher surface area materials do not always prove to be the most active, and nor is the copper surface area across the range of oxides always directly proportional to the activity. We can extend these conclusions to account for the post‐reaction surface area losses. It seems that different preparation conditions, methods, and secondary oxides strongly influence the rate of initial deactivation of the catalysts, which is a key factor in their activity after stabilisation.

## Experimental Section

### Materials

Copper(II) acetate monohydrate (puriss. p. a., ≥99.0 %), zinc(II) acetate dihydrate (puriss. p. a., ≥99.0 %), manganese(II) acetate tetrahydrate (99+%) copper(II) nitrate hemipentahydrate, zinc(II) nitrate hexahydrate, manganese(II) nitrate tetrahydrate, cerium nitrate, magnesium(II) nitrate hexahydrate, sodium carbonate, and cerium acetylacetonate hydrate were all purchased from Sigma–Aldrich. Magnesium(II) acetate tetrahydrate (analytical) was obtained from Amresco. Ethanol (absolute 99.8 %, Certified AR) was purchased from Fischer Scientific and CO_2_ (CP grade) was provided by BOC. All purchased materials were used as received. Deionised water was provided in‐house.

### Co‐precipitated catalysts

The co‐precipitated catalyst precursors were synthesised by co‐precipitation of metal salts using a Toledo Metrohm autotitrator.

Copper nitrate hemipentahydrate (Cu(NO_3_)_2_⋅2.5 H_2_O), zinc nitrate hexahydrate (Zn(NO_3_)_2_⋅6 H_2_O), and aluminium nitrate nonahydrate (Al(NO_3_)_2_⋅9 H_2_O) were dissolved in deionised water to create a mixed‐metal solution with a total molar concentration of 0.25 M. Additionally, a base solution was created by dissolving sodium carbonate (Na_2_CO_3_) in deionised water to give a concentration of 1.5 m Na_2_CO_3_.

A small aliquot (20 cm^3^) of the mixed metal solution was added to the reaction vessel, which was stirred continuously. The amount of liquid was chosen such that it was sufficient to cover the pH probe. This initial aliquot was brought to pH 6.5 by the addition of the base solution until the target pH was reached.

Subsequently, the mixed metal solution was added to the vessel at a rate of 5 cm^3^ min^−1^ with continuous stirring. Concurrently, base solution was added at a sufficient rate to ensure that the reaction mixture maintained a constant pH of 8. Once all of the mixed metal solution was added, the pH was monitored and controlled for a further 10 min to ensure complete precipitation of the material. Thereafter, the precipitate was allowed to age in solution at 65 °C for 3 h.

This precipitate was filtered under suction and washed with water to remove excess sodium salts. This material was then dried at 110 °C for 16 h before being calcined at 325 °C (thermal ramp rate 5 °C min^−1^) in static air for 3 h to produce 2.5–3 g of the catalyst.

### Supercritical anti‐solvent precipitation catalysts

A mixed solution of Cu(OAc)_2_⋅H_2_O (4.1561 mg mL^−1^) with either Zn(OAc)_2_⋅2 H_2_O (1.9584 mg mL^−1^), Mg(OAc)_2_⋅4 H_2_O (1.9132 mg mL^−1^), Mn(OAc)_2_⋅4 H_2_O (2.1866 mg mL^−1^), or Ce(acac)_3_⋅*x* H_2_O (3.9026 mg mL^−1^) was prepared in a 5 vol % H_2_O/ethanol mixture (1000 mL) to give a nominal Cu:X molar ratio of 70:30. SAS preparation was performed using apparatus manufactured by Separex. Liquefied CO_2_ was pumped to give a flow rate of 6.5 kg h^−1^ and the whole system was pressurised to 110 bar and held at 40 °C. Initially, pure solvent (5 vol % H_2_O/ethanol) was pumped through the fine capillary into the precipitation vessel, with a flow rate of 6.5 mL min^−1^ for 15 min, in co‐current mode with scCO_2_ in order to obtain steady state conditions inside the vessel. After this initial period, the flow of liquid solvent was stopped and the mixed metal solution was delivered at a flow rate of 6.5 mL min^−1^. This gave a scCO_2_/mixed metal solution molar ratio of 22:1. The system pressure and temperature were maintained and the preparation conditions were carefully controlled. Leak checks were also periodically carried out throughout the procedure using snoop solution. When all the mixed‐metal solution had been processed, a drying step was carried out. This was achieved by pumping pure ethanol at 6.5 mL min^−1^ co‐currently with scCO_2_ for 30 min, before leaving with just scCO_2_ to pump for a further 60 min. This was to wash the vessel in case residual solvent condensed during depressurisation and partly solubilised the prepared materials. When the drying step was complete the scCO_2_ flow rate was stopped, the vessel was depressurised to atmospheric pressure and the precipitate was collected. Experiments were conducted for approximately 5 h which resulted in the synthesis of ca. 2.5–3 g of solid.

### XRD

Powder X‐ray diffraction measurements were performed using a PANalytical X′pert Pro diffractometer with Ni filtered CuKα radiation source operating at 40 kV and 40 mA. Patterns were recorded over the range of 10–80° 2θ using a step size of 0.016°. All patterns were matched using the ICDD database.

### Surface area measurements

Cu surface area analysis was carried out on a Quantachrome ChemBET chemisorption analyser equipped with a thermal conductivity detector (TCD). Calcined samples (50 mg) were reduced to catalysts using 10 % H_2_/Ar (30 mL min^−1^) with heating to 140 °C at 10 °C min^−1^, and then to 225 °C at 1 °C min^−1^. For Cu surface area analysis, catalysts were cooled to 65 °C under He for N_2_O pulsing. 12 N_2_O pulses (113 μl each) were followed with 3 N_2_ pulses for calibration. The amount of N_2_ emitted was assumed to amount to half a monolayer coverage of oxygen and that the surface density of Cu is 1.47×1019 atoms m^−2^.

### Catalytic methanol synthesis

The catalytic performance of the catalysts for CO_2_ hydrogenation was determined in a fixed‐bed continuous‐flow reactor. The catalyst (0.2 g, 425–600 μm) was placed in a stainless steel tube reactor with an internal diameter of 4.57 mm. Prior to the reaction, the catalysts were prereduced in a flow of 5 % H_2_/He (30 mL min^−1^) for 1 h at 225 °C under atmospheric pressure. The reactor was then allowed to cool to room temperature before gas flow was switched to the reactant mixture (CO_2_:H_2_:N_2_ 20:60:20 molar %). The pressure was increased to 20 bar using a backpressure regulator before the flow was set to 12.5 mL min^−1^ to give a GHSV of 1000 h^−1^. The reactions were conducted at 225 °C. All post‐reactor lines and valves were heated at 110 °C to avoid product condensation. The gas products were analysed via online gas chromatography using an Agilent 7890 system with a flame ionisation detector (FID) and TCD. Nitrogen was used as an internal standard. Samples were taken every 15 min over the course of 8 h. CO_2_ conversion was calculated by the change in moles of CO_2_ compared to calibration runs. The selectivities of methanol and CO represent the respective carbon molar % of the products. In all cases, methanol and CO were the only products observed.

After the reaction, the reactor was depressurised and left under flowing helium (10 mL min^−1^) until cool. The catalyst was then recovered from the tubes and subjected to another set of copper surface area measurements as above. The reduction step was performed again to minimise the effects of passivation caused by contact with air during transit.

## Conflict of interest

*The authors declare no conflict of interest*.
